# Revision of the Australian genus *Logasa* Chandler (Staphylinidae, Pselaphinae, Faronitae) with description of three new species

**DOI:** 10.3897/zookeys.886.39446

**Published:** 2019-11-05

**Authors:** Jun-Young Kang, Donald S. Chandler, Jong-Seok Park

**Affiliations:** 1 Chungbuk National University, 1 Chungdae-ro, Seowon-gu, Cheongju-si, Chungbuk-do 28644, South Korea Chungbuk National University Cheongju South Korea; 2 Department of Biological Sciences, University of New Hampshire, Durham, NH 03824, USA University of New Hampshire Durham United States of America

**Keywords:** beetle, biodiversity, biogeography, systematics, taxonomy

## Abstract

The Australian genus *Logasa* Chandler, 2001 (type species: *L. novaeanglia* Chandler) is comprised of six species, three of which are described as new: *Logasa newtoni***sp. nov.**, *Logasa thayerae***sp. nov.**, and *Logasa comforti***sp. nov.** Examination of the types of *L. tricolor* (Oke) and *L. ventralis* (Oke) revealed that they do not share some of the diagnostic characters used here to characterize the type, and other species of the genus, but they are retained in *Logasa* until the group is revised. A key to species, illustrations of their habitus, and diagnostic characters are provided.

## Introduction

The Australian genus *Logasa* Chandler was described in 2001 based on *Logasa novaeanglia* Chandler. Two other species, *L. tricolor* (Oke, 1928) and *L. ventralis* (Oke, 1928), were originally described in the genus *Sagola* (Oke, 1928), and were transferred to *Logasa* by Chandler (2001). During our revisionary study based on 140 specimens, three new species were recognized. After examination of the types of *L. tricolor* and *L. ventralis*, we found that at the generic level they have different useful diagnostic characters that are based on their foveal system and the form of the male genitalia. This combination of features has not been seen in any of the existing known faronite genera. The Australian faronite fauna has numerous undescribed species (Park and Chandler 2017), which will become the targets of further studies of this very rich fauna. In this study, *L. tricolor* and *L. ventralis* are retained in the genus *Logasa*, but are not further treated here as they may be placed in a new genus once a subsequent revisionary study is conducted with the discovery of additional specimens of these two species.

## Materials and methods

One hundred and forty specimens were examined. They are deposited in the following collections:

**ANIC** Australian National Insect Collection, Canberra, ACT, Australia;

**CBNUIC** Chungbuk National University Insect Collection, Cheongju, South Korea;

**FMNH** Field Museum of Natural History, Chicago, IL, USA;

**MV** Museum of Victoria, Melbourne, Victoria, Australia;

**QM** Queensland Museum, South Brisbane, Queensland, Australia;

**UNHC** University of New Hampshire Insect Collection, Durham, NH, USA.

One specimen of each species was mounted on a permanent microscope slide to observe the internal characters and fine external characters that are not apparent when using a dissecting microscope. Permanent microscopic slides were prepared using the techniques described by Hanley and Ashe (2003). Terminology and nomenclature for the descriptions follow Chandler (2001). Paired structures such as fovea are treated as singular. The morphological right and left of parameres refer to orientation on the illustrations. Decimal degrees were used for the format of geographical coordinates. Holotypes are deposited in ANIC, and paratype depositions are indicated parenthetically. Specimen label data for the holotypes is transcribed verbatim. Data for paratypes are standardized for consistency. The map of Australia is based on an image from SimpleMappr (Shorthouse 2010) that was subsequently modified to add locality marks.

## Systematics

### Family Staphylinidae Latreille, 1802

#### Subfamily Pselaphinae Latreille, 1802

**Supertribe Faronitae Reitter, 1882**

##### Genus Logasa

Taxon classificationAnimaliaColeopteraStaphylinidae

Chandler, 2001

54C2ECB9-726C-568A-93BC-25E33DA5CE1D

http://zoobank.org/14006A6B-F528-41B1-94FF-03871A4617C5

Logasa Chandler, 2001: 47.

###### Type species.

*Logasa novaeanglia* Chandler (designated by Chandler 2001: 47).

###### Diagnosis.

**Figure 1.**
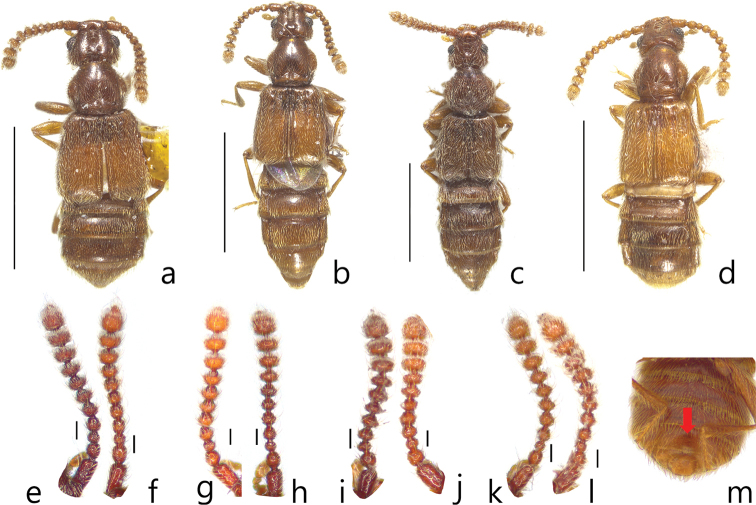
Habitus figures, dorsal view **a***Logasa novaeanglia* Chandler **b***L. newtoni* sp. nov. **c***L. thayerae* sp. nov. **d***L. comforti* sp. nov. Antennae *L. novaeanglia* Chandler: **e** male **f** female *L. newtoni* sp. nov.: **g** male **h** female *L. thayerae* sp. nov.: **i** male **j** female *L. comforti* sp. nov.: **k** male **l** female. Male abdominal venter of *L. comforti* sp. nov. **m** setose depression (arrow). Scale bars: 1 mm (**a–d**), 0.1 mm (**e–l**).

**Figure 2.**
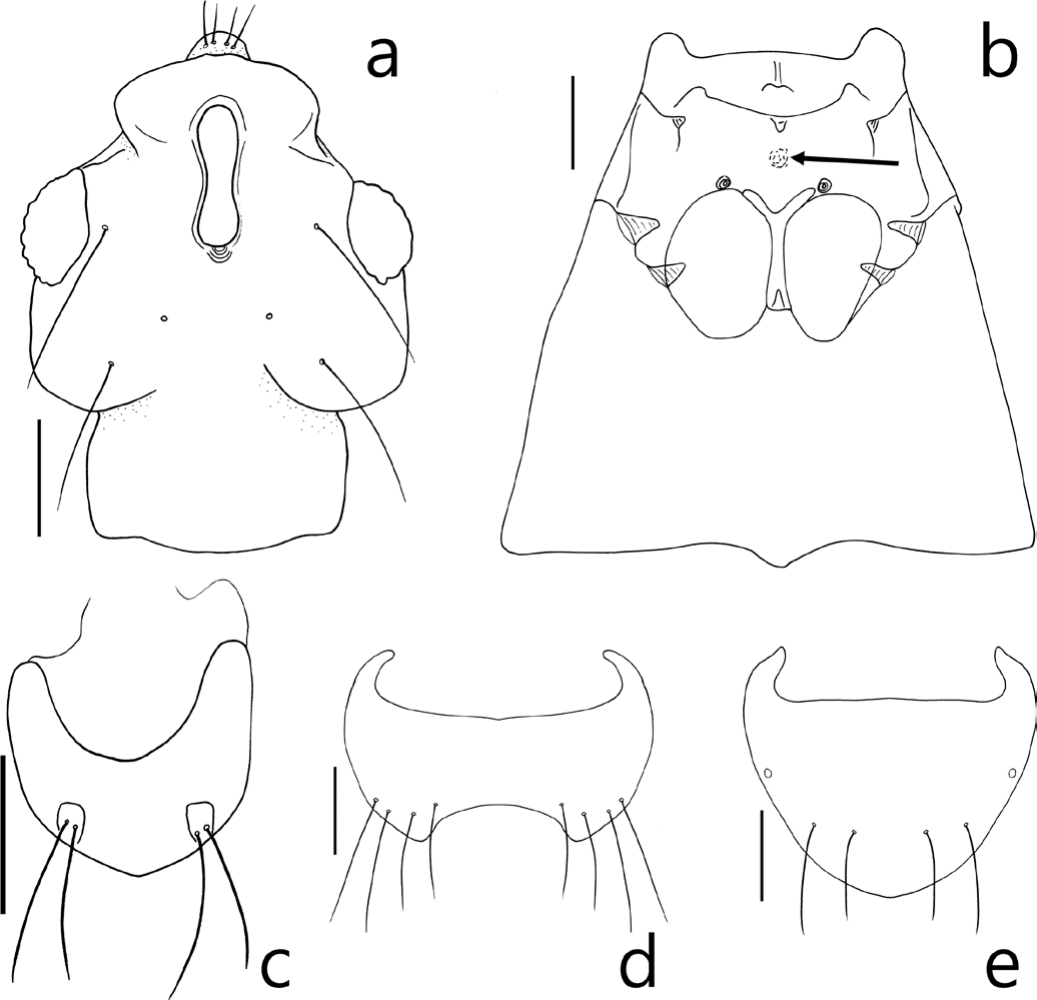
*Logasa newtoni* sp. nov. **a** head, dorsal view **b** meso- and metaventrite, ventral view **c** female abdominal ventrite IX, ventral view **d** male abdominal ventrite VIII, ventral view **e** male abdominal tergite VIII, dorsal view. Scale bar: 0.1mm.

**Figure 3.**
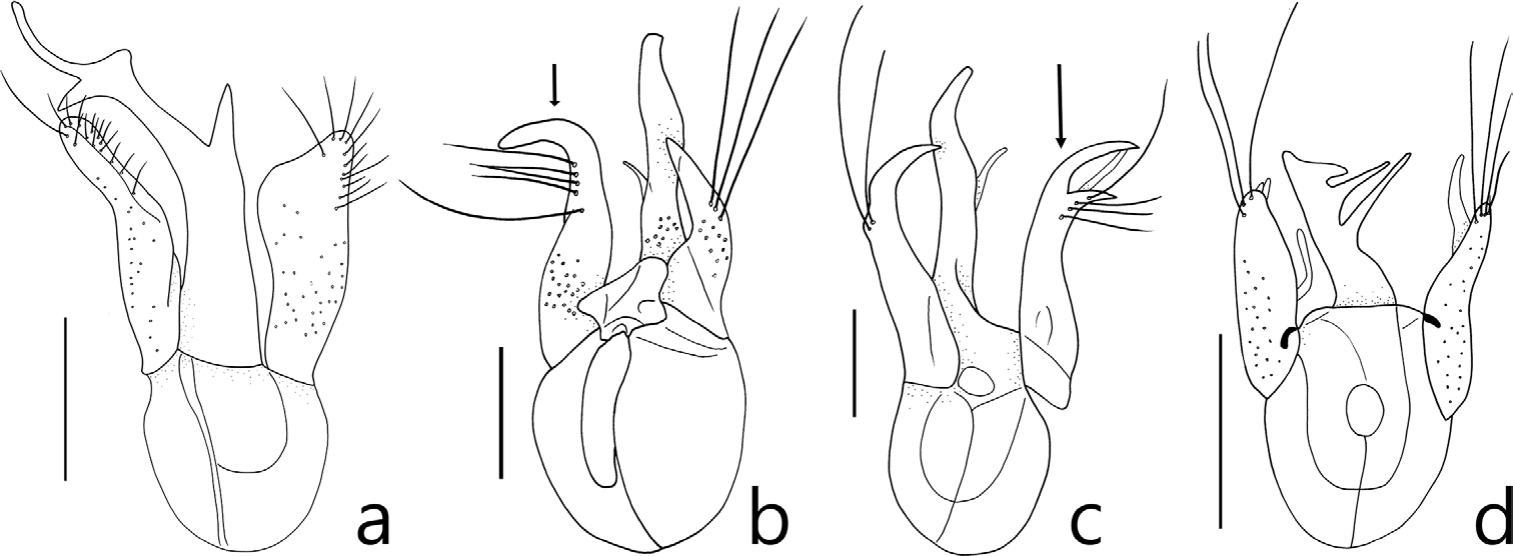
Aedeagi, dorsal view **a***Logasa novaeanglia* Chandler **b***L. newtoni* sp. nov. **c***L. thayerae* sp. nov. **d***L. comforti* sp. nov. Scale bar: 0.1 mm.

Members of this genus are easily separated from other faronite genera by the following combination of characters: head with long frontal sulcus, closed anteriorly (Fig. 2a); elytra rectangular and longer than wide, hind wings fully developed (Fig. 1a–d); mesoventrite with lateral mesosternal fovea, promesocoxal fovea, and lateral mesocoxal fovea (Fig. 2b), and round setal patch at center (Fig. 2b: arrow); metaventrite with metasternal fovea and median metasternal fovea (Fig. 2b); male abdominal ventrite VIII with setose depression (Fig. 1m) located at middle; abdominal ventrites without basolateral fovea; length of abdominal ventrites and tergites VI–VII longer than others (Fig. 1a–d); female abdominal ventrite IX with two pairs of long setae (Fig. 2c); parameres of male genitalia asymmetric and shorter than apical lobe (Fig. 3a–d); phallobase of median lobe rounded and asymmetric (Fig. 3a–d).

###### Distribution.

Southeast Australia (New South Wales, Victoria, Tasmania).

###### Comments.

All specimens, both male and female, of the genus *Logasa* have fully-developed hind wings, and many specimens were collected by flight intercept traps. Members of this genus are usually found in temperate or rainforest habitats in southeastern Australia. Male specimens have a setose depression located at the middle of abdominal ventrite VIII as a secondary sexual character, but this is not easily observed on some specimens. Abdominal ventrite IX is important for recognizing the sex of specimens: in males ventrite IX is usually fragile and partially concealed by ventrite VIII; in females it is more robust, triangular and bears a pair of long setae (Park and Carlton 2014).

#### Key to species of the genus *Logasa* Chandler

The key is mainly based on male genitalia because most species are indistinguishable based on external morphology.

**Table:** 

1	Antennomeres II and IV subquadrate (Fig. 1i–j); right paramere of male genitalia divided into two lobes (Fig. 3c, long arrow); found in Tasmania and Victoria (Fig. 4: triangle)	***Logasa thayerae* sp. nov.**
–	Antennomeres II and IV longer than wide (Fig. 1e–h, k–l); right paramere of male genitalia not divided (Fig. 3a–b, d)	**2**
2	Apical portion of median lobe of male genitalia not branched (Fig. 3b); left paramere of male genitalia curved to left (Fig. 3b, short arrow); found in New South Wales and Victoria (Fig. 4: circle)	***L. newtoni* sp. nov.**
–	Apical portion of median lobe of male genitalia branched (Fig. 3a, d); left paramere of male genitalia less curved (Fig. 3a, d)	**3**
3	Apical lobe of male genitalia forked into two lobes, major lobe longer than three branches (Fig. 3a); right paramere of male genitalia twice as wide as left (Fig. 3a); found in New South Wales (Fig. 4: square)	***L. novaeanglia* Chandler**
–	Apical lobe of male genitalia with three branches extending from right side (Fig. 3d); right paramere of male genitalia slightly broader than left or as wide as left (Fig. 3d); found in Tasmania and Victoria (Fig. 4: diamond)	***L. comforti* sp. nov.**

##### Logasa novaeanglia

Taxon classificationAnimaliaColeopteraStaphylinidae

Chandler, 2001

52CB3C35-9E23-505D-8986-6AC6BD276E12

http://zoobank.org/CC8FB01A-83D3-4674-8D98-BF2E4441FAE7

Logasa novaeanglia Chandler, 2001: 49.

###### Type material examined.

***Paratypes*** (*N* = 16; 5 males, 11 females). **Australia: New South Wales (NSW)**: 1♂ (aedeagus dissected and placed in micro-vial, UNHC), New England National Park, Wright’s Lookout Trail, 1300 m, 27 II–6 III 1980, *Nothofagus moorei* rainforest, A. Newton, M. Thayer, window trap; 1♂ (UNHC), 1320 m, 15–27 II 1993, D. S. Chandler, FIT, cool temperate rainforest; 1♀ (UNHC), 2–17 IV 1993, D. S. Chandler, FIT, cool temperate rainforest; 1♀ (UNHC), 28 II–14 III 1993, D. S. Chandler, FIT, cool temperate rainforest; 1♀ (UNHC), 1330 m, 17 V 1993, D.S. Chandler, *Nothofagus moorei* leaf litter; 2♂♂ 2♀♀ (1♂ 2♀♀ in UNHC, 1♂ in QM), Styx River State Forest, Cedar Pit Floral Reserve, 42 km south east Wollomombi, 935 m, 25 II–15 III 1993, D.S. Chandler, FIT, old temperate rainforest; 1♂ (UNHC), 16 III–4 IV 1994, D.S. Chandler, FIT, old temperate rainforest; 1♀ (UNHC), 3–15 II 1993, K. MacGregor, FIT, old temperate rainforest; 1♀ (ANIC), 20 IV–12 V 1993, D.S. Chandler, FIT, old temperate rainforest; 1♂ (UNHC), 40 km south east Wollomombi, 990 m, 16 III–4 IV 1993, D.S. Chandler, FIT, old wet sclerophyll; 1♀ (ANIC), 2–14 XII 1993, K. MacGregor, FIT, old wet sclerophyll; 1♀ (ANIC), 6 XI–1 XII 1993, K. MacGregor, FIT, old wet sclerophyll; 1♀ (ANIC), 25 II–15 III 1993, D.S. Chandler, FIT, old wet sclerophyll; 1♀ (ANIC), 15 X–5 XI 1993, K. MacGregor, FIT, old wet sclerophyll.

###### Additional materials

(*N* = 26; 10 males, 16 females). **Australia: NSW**: 6♂♂ 14♀♀ (2♂ aedeagus dissected and mounted in Euparal on clear plastic card, FMNH), New England National Park, Robinson’s Knob Road, 1 km east Park Gate, 1320 m, 30.30S, 152.24E, 29 XII 1986–14 I 1987, *Nothofagus moorei* forest, A. Newton & M. Thayer 781, FMHD#86-689, FIT & window; 1♂ (FMNH), 1305 m; 30.30S, 152.24E, 29 XII 1986, *Nothofagus moorei* forest, A. Newton & M. Thayer 780, FMHD#86-688, berlese, leaf & log litter, forest floor; 3♂♂ 2♀♀ (CBNUIC), 29 XII 1986–14 I 1987, *Nothofagus moorei* forest, A. Newton & M. Thayer 780, FMHD#86-686, flight intercept (window) trap.

###### Diagnosis.

This species can be distinguished from the other species of the genus *Logasa* by the following combination of characters: antennomeres II and IV rectangular and longer than wide (Fig. 1e–f), apical lobe of male genitalia forked into two lobes, major lobe longer with three branches, parameres with over ten setae, and right lobe shorter and wider (Fig. 3a).

###### Description.

Length 2.1–2.5 mm. Body yellowish to reddish-brown (Fig. 1a). *Head.* Head triangular with frontal fovea and vertexal foveae. Antennomeres with tubercles and long setae (Fig. 1e–f). Antennomere I elongate, II rectangular, III subquadrate and smallest, IV rectangular, V rhombic, VI—X gradually transverse (Fig. 1e–f). *Thorax.* Pronotum with deep sulcus and pair of lateral antebasal foveae. Each elytron with basal elytral foveae and discal elytral foveae. *Abdomen*. Tergite IV with pair of transverse patches of microtrichia (Fig. 1a). *Aedeagus.* Apical lobe of male genitalia forked into two lobes, major lobe longer with three branches, minor lobe short and simple (Fig. 3a). Phallobase of median lobe asymmetric and rounded (Fig. 3a). Parameres asymmetric with over ten setae, left paramere longer and narrower than right (Fig. 3a).

###### Distribution.

**Figure 4.**
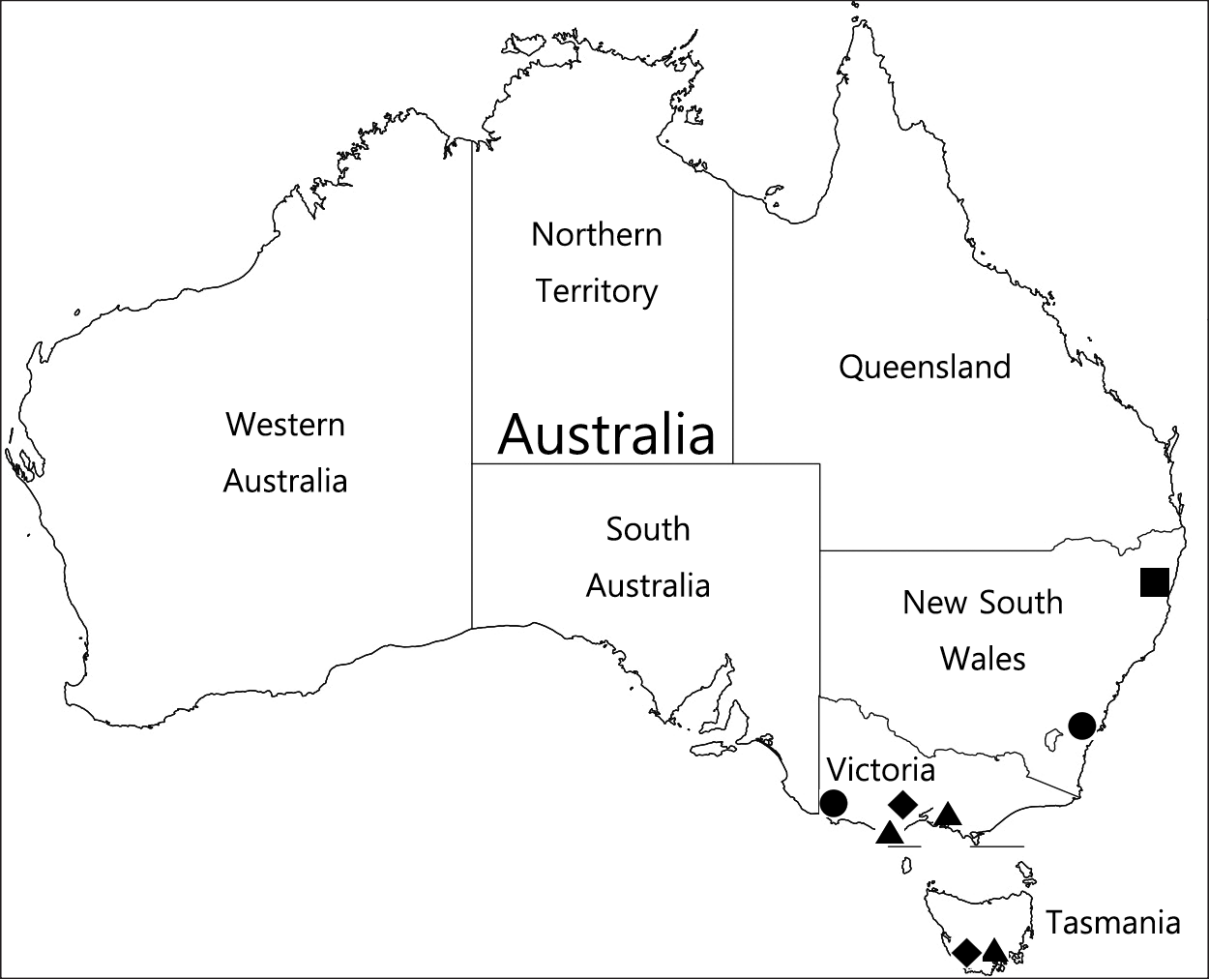
Collection localities of *Logasa novaeanglia* Chandler: square *L. newtoni* sp. nov.: circle *L. thayerae* sp. nov.: triangle *L. comforti* sp. nov.: diamond.

New South Wales (Fig. 4: square).

###### Habitat.

Specimens of this species were collected using flight intercept traps, window traps, or by sifting leaf and log litter in wet sclerophyll forests.

##### Logasa newtoni

Taxon classificationAnimaliaColeopteraStaphylinidae

Kang, Chandler & Park sp. nov.

E068AD95-CCAD-5C82-A214-FC96738D9C75

http://zoobank.org/66785C9A-2DA5-4593-A9EA-BB78B2DA22AA

###### Type material.

***Holotype*. Australia: NSW**: ♂ (ANIC), “AUSTRALIA: NSW., / Brown Mtn. Floral / Res., 0.5 km SSW / Cochrane Dam, 940 m” “II – 8/ 22 – 1993 / A Newton & M Thayer / cool temp. rainfor / window trap”. ***Paratypes*** (*N* = 83; 25 males, 58 females). **Australia: NSW**: 1♂2♀♀ (FMNH), Brown Mountain Flora Reserve, 0.5 km SSW Cochrane Dam, 950 m, 36.35S, 149.27E, 20 XII 1986, warm-temperate rainforest, A. Newton & M. Thayer 767, FMHD #86-650, berlese, leaf & log litter, forest floor; 6♂♂ 41♀♀ (3♂♂ aedeagus dissected and mounted in Euparal on clear plastic card, 1♀ slide mounted, FMNH), 20 XII 1986–15 II 1987, warm-temperate rainforest, A. Newton & M. Thayer 767, FMHD#86-648, FIT & window; 13♂♂ 12♀♀ (1♂ aedeagus dissected and mounted in Euparal on clear plastic card, 1♂ slide mounted, 1♂ 2♀♀, CBNUIC; 10♂♂ 10♀♀, UNHC), 940 m, 8–22 II 1993, A. Newton & M. Thayer, cool temperate rainforest, window trap; 3♀♀ (UNHC), Brown Mountain, 853 m, 30 III 1967, rainforest, RWT, RJB, ANIC Berlesate No. 20 leafmould; 1♂ (aedeagus dissected and placed in micro-vial, left paramere damaged, ANIC), ca. 914 m, 9 XII 1967, rainforest, Taylor & Brooks, ANIC Berlesate No. 42 leafmould; 1♂ (aedeagus dissected and placed in micro-vial, left paramere damaged, ANIC), Rutherford Creek, 9 I 1968, rainforest, M. Upton, ANIC Berlesate No. 55 leafmould; 1♂ (ANIC), nr Nimmitabel, Rutherford Creek, 910 m, 26 V 1970, RWT & R. Bartell, rainforest, ANIC Berl. #287; **Victoria (VIC)**: 1♂ (aedeagus dissected and mounted in Euparal on clear plastic card, MV), Road C187, 15 km west Dartmoor, 1 VIII 2012, ground moss, blackwood stringybark forest.

###### Diagnosis.

This species can be distinguished from the other *Logasa* species by the following combination of characters: antennomeres II and IV rectangular and longer than wide (Fig. 1g–h), apical lobe of male genitalia not divided, left paramere of male genitalia curved to left (Fig. 3b).

###### Description.

Length 1.6–2.5 mm. Body yellowish to reddish-brown (Fig. 1b). *Head.* Head triangular with frontal fovea and vertexal foveae. Antennomeres with tubercles and long setae (Fig. 1g–h). Antennomere I elongate, II rectangular, III subquadrate and smallest, IV rectangular, V—VII rhombic, VIII—X gradually transverse (Fig. 1g–h). *Thorax.* Pronotum with deep sulcus and pair of lateral antebasal foveae. Each elytron with basal elytral foveae and discal elytral foveae. *Abdomen*. Tergite IV with pair of transverse patches of microtrichia (Fig. 1b). *Aedeagus.* Apical lobe of male genitalia not branched (Fig. 3b). Phallobase of median lobe asymmetrical and rounded (Fig. 3b). Parameres of male genitalia asymmetrical, left longer than right and curved to left (Fig. 3b).

###### Distribution.

New South Wales, Victoria (Fig. 4: circle).

###### Etymology.

This species is named for one of the co-collectors of the holotype and world-renowned beetle specialist, Alfred F. Newton.

###### Habitat.

Most specimens of this species were collected using flight intercept traps, window traps, or by sifting leaf and log litter in temperate forest or rainforest habitats.

##### Logasa thayerae

Taxon classificationAnimaliaColeopteraStaphylinidae

Kang, Chandler & Park sp. nov.

E149EFC1-F67D-5FB4-88BD-1D654C52EDEF

http://zoobank.org/E70F5324-AF5F-4A20-9B1D-DC613B4A08FB

###### Type material.

***Holotype*. Australia: VIC**: ♂ (MV), aedeagus dissected in micro-vial, “AUSTRL.: VIC.: Otway N.P., / Malts Rest, 260 m / 38.45S 143.33E / 25.I–8.II.1987 / wet scleroph. - *Noth. cunn*.” “A. Newton & M. Thayer 807 / FMHD #87-206 / flight intercept / (window) trap”. ***Paratypes*** (*N* = 6; 2 males, 4 females). **Australia: VIC**: 1♂ 2♀♀ (1♂ aedeagus dissected and whole body placed in micro-vial, 1♀ slide mounted, UNHC), 10 km east of Maryville, 27 XI 1986, D. Burckhardt; 1♀ (UNHC), Belgrave, V.F.E. Wilson, I 1922, fallen leaves (MV); **Australia: Tasmania (TAS)**: 1♂ (aedeagus dissected and whole body placed in micro-vial, MV), Zeehan, 16 IV 1895; 1♀ (MV), Mt. Wellington, I 1948, C. Oke.

###### Diagnosis.

This species can be distinguished from the other *Logasa* species by the following combination of characters: antennomeres II and IV subquadrate (Fig. 1i–j), apical lobe of male genitalia not divided, left paramere of male genitalia curved to right, and right paramere divided into two lobes apically and curved to right (Fig. 3c).

###### Description.

Length 2.0–2.3 mm. Body yellowish to reddish-brown with long setae (Fig. 1c). *Head*. Head triangular with frontal fovea and vertexal foveae. Antennomeres with tubercles and long setae (Fig. 1i–j). Antennomere I elongate, II–IV subquadrate, III smallest, V–VI rhombic, VII—X gradually transverse (Fig. 1i–j). *Thorax.* Pronotum with the deep sulcus and pair of lateral antebasal foveae. Each elytron with basal elytral foveae and discal elytral foveae. *Abdomen*. Tergite IV with patches of microtrichia (Fig. 1c). *Aedeagus.* Apical lobe of male genitalia not branched (Fig. 3c). Phallobase of median lobe asymmetrical and rounded (Fig. 3c). Left parameres of male genitalia curved to right (Fig. 3c). Right paramere divided into two lobes apically and curved to right (Fig. 3c).

###### Distribution.

Tasmania, Victoria (Fig. 4: triangle).

###### Etymology.

This species is named for one of the co-collectors of the holotype and world-renowned beetle specialist, Margaret K. Thayer.

###### Habitat.

Most specimens of this species were collected using flight intercept (window) traps in wet sclerophyll forests.

##### Logasa comforti

Taxon classificationAnimaliaColeopteraStaphylinidae

Kang, Chandler & Park sp. nov.

9DC439E2-AFA6-59EC-A8F9-6ED0B229E5B2

http://zoobank.org/5268E63A-440F-4E4B-B861-078605AA90E3

###### Type material.

***Holotype*. Australia: TAS**: ♂ (ANIC), “43.25S 146.10E TAS / Melaleuca near / Bathurst Harbour / 15 Apr.–29 May 1991 / M. Comfort F.I.T.#1”, “F.I.T. / ANIC 1185 / closed forest”. ***Paratypes*** (*N* = 6; 3 males, 3 females). **Australia: TAS**: 1♂ 1♀ (ANIC), Melaleuca near Bathurst Harbour, 43.25S, 146.10E, 15 IV–29 V 1991, M. Comfort, FIT#1, ANIC 1185, closed forest; 1♀ (ANIC), 29 V–29 VIII 1991, M. Comfort, FIT#1, ANIC 1190, closed forest; 1♂ (aedeagus dissected and mounted in Euparal on clear plastic card, ANIC), 29 VIII–28 XI 1991, I. Naumann & G. Clarke, FIT#1, closed forest, ANIC 1202; 1♀ (ANIC), 15 III–15 IV 1991, E. Edwards, J. Berry, FIT#1, ANIC 1179, closed forest; **VIC**: 1♂ (aedeagus dissected in micro-vial, MV), Ballarat, C. Oke.

###### Diagnosis.

This species can be distinguished from the other *Logasa* species by the following combination of characters: antennomeres II and IV rectangular and longer than wide (Fig. 1k–l), apical lobe of male genitalia with three branches bearing to right, left paramere broader than right (Fig. 3d).

###### Description.

Length 1.6–1.9 mm. Body yellowish to reddish-brown (Fig. 1d). *Head.* Head triangular with frontal fovea and vertexal foveae. Antennomeres with tubercles and long setae (Fig. 1k–l). Antennomere I elongate, II rectangular, III subquadrate, IV rectangular, V–VI rhombic, VII–X gradually transverse (Fig. 1k–l). *Thorax.* Pronotum with deep and distinct pit in the middle and lateral antebasal foveae. Each elytron with basal elytral foveae and discal elytral foveae. *Abdomen*. Tergite IV with patches of microtrichia (Fig. 1d). *Aedeagus.* Apical lobe of male genitalia with three branches bearing to right (Fig. 3d). Phallobase of aedeagus asymmetric and rounded (Fig. 3d). Left paramere broader than right (Fig. 3d).

###### Distribution.

Tasmania, Victoria (Fig. 4: diamond).

###### Etymology.

This species is named for the collector of the holotype, C. Comfort.

###### Habitat.

Most specimens of this species were collected using flight intercept traps in forests.

## Supplementary Material

XML Treatment for Logasa

XML Treatment for Logasa novaeanglia

XML Treatment for Logasa newtoni

XML Treatment for Logasa thayerae

XML Treatment for Logasa comforti
